# Improving the Electrochemical and Electrochromic Properties of Copolymerized 3,4-Ethylenedioxythiophene with Pyrene

**DOI:** 10.3390/polym17010069

**Published:** 2024-12-30

**Authors:** Xiang Wang, Haiyun Jiang, Muling Gan, Jun Zhang, Ruomei Wu, Weili Zhang, Ziyi Wang, Minxi Guo, Yangfan Mu

**Affiliations:** 1 School of Packaging and Materials Engineering, Hunan University of Technology, Zhuzhou 412007, China; xiangwang1345@163.com (X.W.); 15292278813@163.com (M.G.); 18205528834@163.com (J.Z.); cailiaodian2004@126.com (R.W.); zh_weili@163.com (W.Z.); w15377559340@163.com (Z.W.); gmxydhfc@163.com (M.G.); 18585843461@163.com (Y.M.); 2 National & Local Joint Engineering Research Center for Advanced Packaging Material and Technology, Zhuzhou 412007, China

**Keywords:** electrochemical copolymerization, copolymers, electrochromic property, redox stability

## Abstract

Pyrene (Pr) was used to improve the electrochemical and electrochromic properties of polythiophene copolymerized with 3,4-ethylenedioxythiophene (EDOT). The corresponding product, poly(3,4-ethylenedioxythiophene-co-Pyrene) (P(EDOT-co-Pr)), was successfully synthesized by electrochemical polymerization with different monomer concentrations in propylene carbonate solution containing 0.1 M lithium perchlorate (LiClO_4_/PC (0.1 M)). The homopolymer and copolymer films were analyzed by Fourier transform infrared spectroscopy (FT-IR), color-coordinate and colorimetric methods, cyclic voltammetry (CV), spectroelectrochemistry (SEC), and UV–visible spectroscopy (UV-Vis). Homopolymer poly(3,4-ethylenedioxythiophene) (PEDOT) and the P(EDOT-co-Pr) copolymer were investigated, which included examining their colorimetric, electrochemical, and electrochromic characteristics. The color shifts resulting from redox reactions of the polymers were also observed. The copolymers with different monomer concentrations achieved multicolor shifts, such as light purple, dark blue, dark red, green, and earthy yellow. Moreover, P(EDOT-co-Pr) had a small optical bandgap (1.74–1.83 eV), excellent optical contrast (31.68–45.96%), and high coloring efficiency (350–507 cm^2^ C^−1^). In particular, P(EDOT1-co-Pr3) exhibited outstanding cycling stability, retaining 91% of its initial optical contrast after cycling for 10,000 s, and it is expected to be a promising candidate copolymer for electrochromic applications.

## 1. Introduction

Electrochromic materials are defined as the occurrence of stable and reversible color changes of their optical characteristics in the presence of an applied voltage, which is manifested in the occurrence of a reversible change in color and transparency [1,2]. Over the past few decades, electrochromic-conjugated polymer materials have attained widespread attention and interest because of their low operating voltage characteristics [3,4,5], fast switching response speed [6,7], rich multicolor transition capability [8,9], and excellent transmittance contrast [10,11]. Because of their excellent properties, electrochromic materials are extensively used in diverse fields such as smart windows [12,13], energy storage devices [14,15], sensors [16,17,18], military camouflage [19,20], and other fields. Designing and improving the optical and electrochromic characteristics of electrochromic materials for commercial applications has been a major focus in recent years [21,22]. Besides these properties, it is also crucial to create multicolor changes through simple and economical synthetic methods. Poly(3,4-ethylenedioxythiophene) (PEDOT) has been extensively investigated due to its excellent properties, such as low bandgap, adjustable hue by chemical structure modification, good stability, and fast response time [23,24,25]. The color transition of PEDOT is usually light to dark blue [26]. Electrochromic properties are closely related to the doping and de-doping processes. Doping alters the electronic configuration of electrochromic materials and generates new electronic states within their bandgaps, which trigger a visible color change. During the doping and de-doping process, the electrochromic materials’ bandgaps change reversibly and the electronic absorption spectra present a shifting phenomenon, which shows a color switch in the visible region [27].

Introducing additional π-conjugated units into the main chain of PEDOT is one of the important methods for changing its bandgap and light-absorbing properties to enrich the color variations. For example, W. Yu et al. electrochemically copolymerized EDOT with 5-cyanoindole, and the copolymer film could shift between violet and blue [28]. M. Guzel et al. electrochemically copolymerized EDOT with a carbazole derivative, and proposed a method for the preparation of electrophilic color-changing materials to achieve the conversion from black to transparent colors [29]. Y. Tao et al. electrochemically copolymerized EDOT with anthracene and achieved multicolor switching of red, green, and blue by controlling the feed ratio [30]. Z. Zhang et al. electrochemically copolymerized EDOT with pyrrole, and achieved multicolor switching of blue, violet, and yellow [31]. H. Zeng et al. electrochemically copolymerized EDOT with bithiophene, and achieved blue-violet to blue-gray switching [32]. Among the methods for preparing electrochromic polymers, electrochemical polymerization is the most simple, convenient, and cost-effective one compared with other chemical synthesis methods [33]. Previously, W. Yang et al. electrochemically copolymerized 4H-cyclopenta[2,1-b:3,4b′] Dithiophene and Pyrene and assembled them into devices to study their electrochromic properties [34]. I. Baltog et al. electrochemically polymerized EDOT and Pyrene in the presence of carbon nanotubes to form composites and studied their optical properties [35]. C. Zhang et al. electrochemically polymerized copolymers of EDOT and Pyrene in tetrabutylammonium perchlorate/acetonitrile (TBAP/ACN) solution and investigated the effects of different feed ratios on their electrochemical and thermal stability properties [36]. However, the effects of different monomer concentrations on the multicolor switching and electrochromic properties of the copolymer films were not systematically investigated.

In this study, we successfully synthesized homopolymer and copolymer films of PEDOT, polypyrene (PPr), and P(EDOT-co-Pr) by electrochemical polymerization. Unlike other experiments, sodium perchlorate/acetonitrile (NaClO_4_/ACN) was not used as the electrolyte in this experiment because the ACN solution volatilizes quickly and requires a closed electrochemical cell. Alternatively, lithium perchlorate/propylene carbonate (LiClO_4_/PC) solution has excellent low volatility and stable electrical resistance, which facilitates the control of electrochromic film formation and reduces the requirement of the preparation environment. Therefore, in this work, LiClO_4_/PC (0.1 M) was used as the electrolyte to prepare homopolymer and copolymer films. Subsequently, we characterized the homopolymer and copolymer films using FT-IR, CV, SEC, UV-Vis, and color-coordinate and colorimetric methods. These characterization methods investigated in detail the structure and electrochemical, and electrochromic properties of the different polymer films. For comparison, the corresponding properties of the homopolymer PEDOT film were analyzed too. The findings indicate that the addition of Pyrene improves the cyclic stability and electrochromic properties of the polymer films, and that films with different monomer concentrations can realize a multicolor switch. These findings provide a novel method for producing high-quality electrochromic copolymer films with reversible multicolor switching ability.

## 2. Materials and Methods

### 2.1. Materials

Pyrene (97%) was purchased from Shanghai McLean Biochemical Co. (Shanghai, China) 3,4-ethylenedioxythiophene (EDOT; AR, 99%), polypropylene carbonate (PC; AR, 99%), and acetonitrile (ACN; AR, 99%) were obtained from Aladdin. Lithium perchlorate (LiClO_4_; AR, 98%) was acquired from Shandong Xiya Chemical Co (Linyi, China). Deionized water was obtained from Watsons (Guangzhou, China). All reagents were commercially available and used as received. The conductive glass coated with indium tin oxide (ITO) was purchased from South China Xiangcheng Technology Co (Yiyang, China). It was washed sequentially with acetone, anhydrous ethanol, and deionized water under ultrasonication and dried at room temperature before use. Shanghai Chuxi Industry Co. (Shanghai, China) provided the platinum (Pt) sheet and silver/silver chloride (Ag/AgCl) electrode that was filled with a saturated potassium chloride solution.

### 2.2. Preparation of Polymer Films

The films prepared in this experiment were made using the following method: polymeric films were prepared by the constant potential method in 60 mL of LiClO_4_/PC (0.1 M) containing different monomer concentrations of EDOT and Pyrene (Table 1). After electrochemical polymerization, the polymer films were washed three times with ACN to exfoliate the electrolytes, oligomers, and monomers. The ITO glass served as the working electrode, a Pt sheet served as the counter electrode, and Ag/AgCl served as the reference electrode in this three-electrode electrochemical preparation system. During the preparation process, the parameters were adjusted to ensure that the total charge through the cell was the same for both homopolymer and copolymer films.

### 2.3. Characterizations

The structure of the homopolymer and copolymer films was investigated using a FT-IR spectrometer (IRTracer-100; SHIMADZU, Kyoto, Japan). Electrochemical tests were performed at room temperature using a PGSTAT302N electrochemical workstation manufactured by Metrohm, Herisau, Switzerland. The electrochemical test system was based on a three-electrode cell structure. The PGSTAT302N electrochemical workstation coupled with a UV-Vis spectrophotometer (UV-2600; SHIMADZU, Japan) was used to investigate the colorimetric, electrochemical, and electrochromic properties of the prepared films. The experimental setup utilized a 10 mm wide colorimetric bath customized for spectrophotometer applications, and LiClO_4_/PC (0.1 M) solution was used as the test electrolyte for the testing process. All experimental procedures were carried out at room temperature.

## 3. Results and Discussion

### 3.1. Polarization Curves and Structural Features

**Figure 1:**
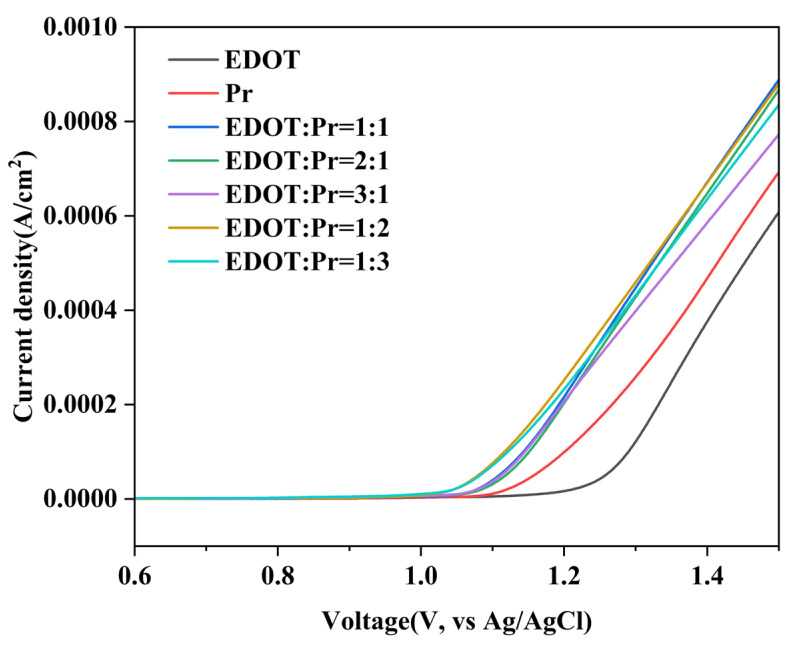
Polarization curves of EDOT, Pyrene, and EDOT/Pyrene in LiClO_4_/PC (0.1 M) solution.

Polarization curves of the EDOT, Pyrene, and EDOT/Pyrene polymers in LiClO_4_/PC (0.1 M) solution at a sweep rate of 50 mV s^−1^ are displayed in Figure 1. For the anodic current (E_pa onset_), the E_pa onset_ of Pyrene is 1.06 V and the onset potential of EDOT is 1.12 V. The difference between the E_pa onset_ of Pyrene and EDOT is only 0.06 V, which suggests that electrochemical copolymerization between the two monomers is readily achievable [37]. This may be due to some changes in the electrochemical environment due to the sequential deposition of the two monomers during the anodic polarization process or due to interactions between the monomers [38,39]. In addition, the lower E_pa onset_ of the copolymers than that of the two monomers suggests successful copolymerization of the two monomers in the LiClO_4_/PC solution.

**Figure 2:**
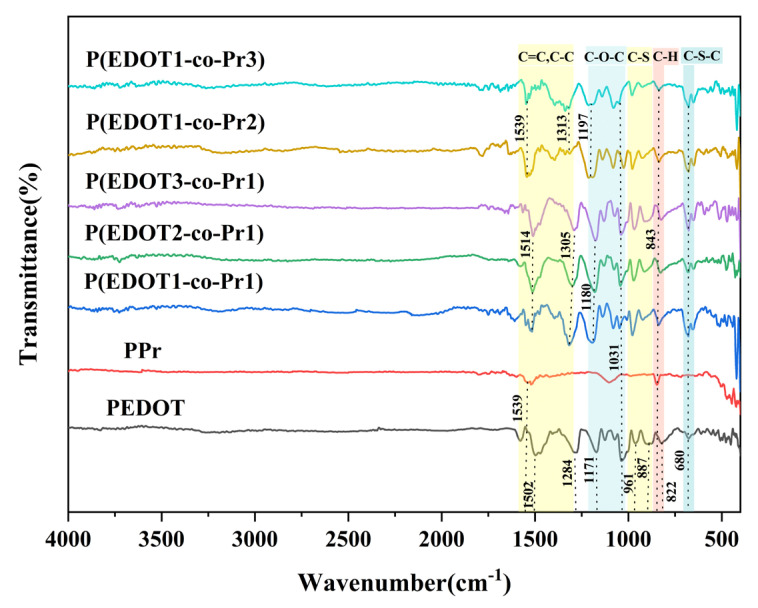
FT-IR spectra of the homopolymer and copolymer films.

Figure 2 displays the corresponding FT-IR spectra of the homopolymer and copolymer films made using different monomer concentrations. According to the PEDOT spectra, the thiophene ring’s C=C and C-C stretching modes produce bands at 1502 and 1284 cm^−1^, while the EDOT ring’s C-O-C and C-S-C stretching modes produce bands at 1171, 1031, and 680 cm^−1^, respectively [32,36,40]. The C-S vibration in EDOT is the source of the bands at 822, 887, and 961 cm^−1^ [41,42]. In the spectrum of PPr, the peak at 843 cm^−1^ is attributed to the out-of-plane bending vibration of the C-H bond of the substituted benzene ring, while the band at 1539 cm^−1^ is associated with the C=C stretching vibration of the Pyrene ring [43]. Compared with the corresponding homopolymers, the copolymer obtained from the EDOT/Pr mixture at 1:1 exhibits a band at 843 cm^−1^, indicating the existence of Pyrene units. A blue shift of the peak at 822, 887, and 961 cm^−1^ is observed, and the peak changes from a wide overlapped double peak to an asymmetrical sharp one. This suggests that Pyrene has been successfully introduced into the EDOT chain. Bands from the EDOT unit were also observed at 1180, 1031, and 680 cm^−1^ [32]. The spectra of the copolymers obtained under 2:1, 3:1, 1:2, and 1:3 conditions also show similar peaks. All these features indicate that the polymers contain both EDOT and Pyrene.

### 3.2. Colorimetric Studies

**Figure 3:**
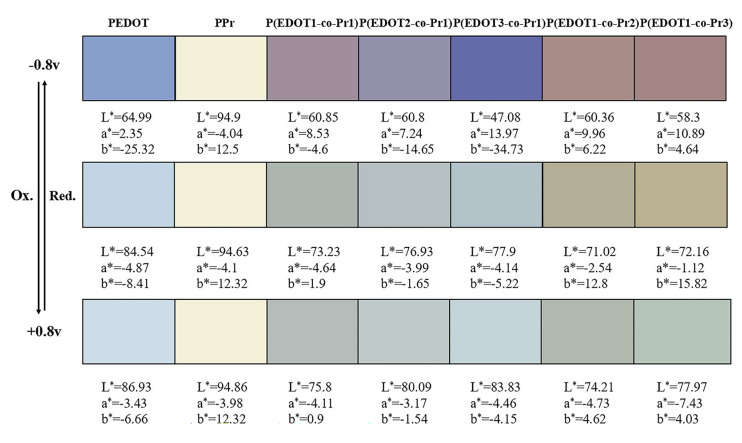
The color coordinates of PEDOT, PPr, P(EDOT1-co-Pr1), P(EDOT2-co-Pr1), P(EDOT3-co-Pr1), P(EDOT1-co-Pr2), and P(EDOT1-co-Pr3) in their reduced, neutral, and oxidized states (at −0.8 v, 0 v, and +0.8 v, respectively).

To regulate the color switching behavior of the conductive polymers, the visible spectral absorption of the homopolymers and copolymers can be regulated by the monomer concentration. Therefore, exploring the effect of different monomer concentrations on the color characteristics of the copolymers is the focus of this study. The CIE 1931 coordinates are obtained by measuring the reflectance of the homopolymer and copolymer films at different wavelengths by UV-Vis spectrophotometry, and its Yxy value is converted to L*a*b* to determine the color coordinates of the homopolymer and copolymer films in the CIE color space. According to the CIE 1931, colors can be accurately described by three parameters: L*, a*, and b* [44]. Figure 3 displays the color changes of the homopolymer and copolymer films in the reduced, neutral, and oxidized states and the values of L*, a*, and b*. All measurements were taken under sunlight at a standard angle (65/2°). The copolymer films at different monomer concentrations present color changes from pink-purple to dark green, light purple to light green, and blue-violet to blue-green, from light red to light brown to green, and from dark red to earthy yellow to green when excited by a gradual potential, unlike the homopolymer PEDOT, which only varies from dark blue to light blue. Notably, the reduction process showed many more color changes of the copolymer films than the oxidation process.

**Figure 4:**
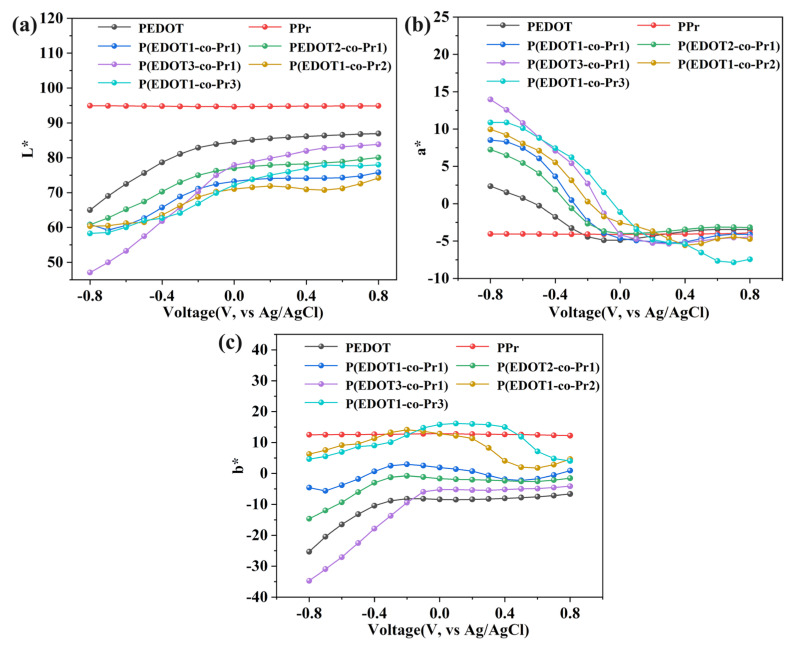
Homopolymers and copolymers from reduced to oxidized states in terms of their (**a**) lightness values, (**b**) a* values, and (**c**) b* values.

When the homopolymer and copolymer films are transferred to an oxidized state, L*, a*, and b* fluctuate, and this is displayed in Figure 4. The L* values show an increasing trend when the copolymer and PEDOT change from a reduced to an oxidized state, which is confirmed by the increase in the brightness of the films. The a* values are shifted to green, and the b* values are shifted to yellow. The higher the Pyrene monomer percentage kept in the copolymer, the more prominently the values shift. The L* value of the copolymer P(EDOT3-co-Pr1) film containing a small amount of Pyrene monomer increases faster, with minimum and maximum values of +47.08 and +83.83, respectively, and the increase in the L* value of the homopolymer PEDOT film is smaller compared with that of the copolymer P(EDOT3-co-Pr1), which is +21.94. At the same time, no change in the L* value of PPr is observed, whose conjugated structure is relatively stable and not prone to inducing significant redox reactions during electrochemical processes. The PPr film has a slower rate of electron transfer due to its low diffusion coefficient.

### 3.3. Electrochemical Performance

**Figure 5:**
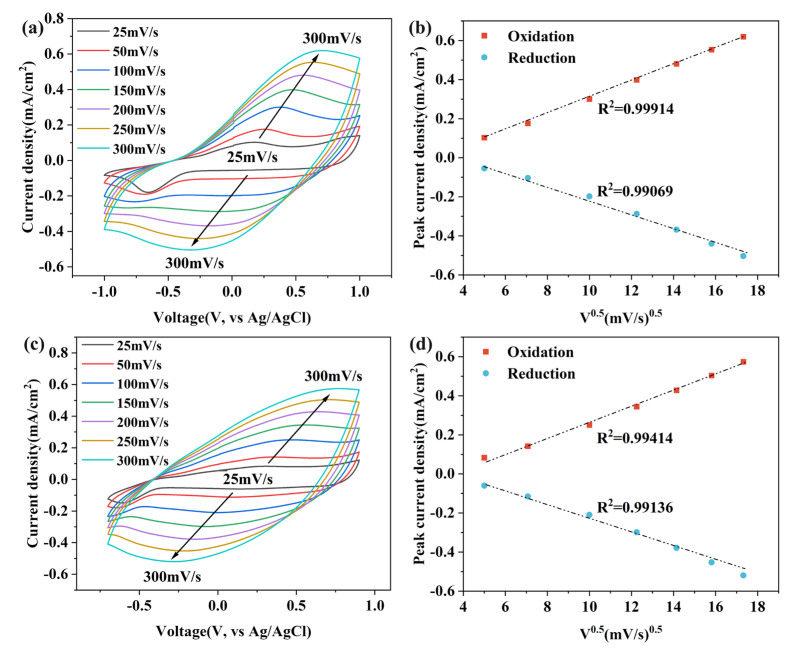
CV curves of (**a**) PEDOT and (**c**) P(EDOT1-co-Pr1) films in monomer-free LiClO_4_/PC (0.1 M) solution at a 25 to 300 mV s^−1^ sweep rate. Electrochemical response of (**b**) PEDOT and (**d**) P(EDOT1-co-Pr1) films.

The electrochemical behavior of the homopolymer and copolymer films was investigated using CV in LiClO_4_/PC (0.1 M) solution devoid of monomers. Since the CV curves of the copolymer films prepared with different monomer concentrations are similar, the P(EDOT1-co-Pr1) film was selected as a representative for discussion. The electrochemical response plots and CV curves of the PEDOT and P(EDOT1-co-Pr1) films at various sweep rates are displayed in Figure 5. The elevated anodic peak potentials and decreased cathodic peak potentials of PEDOT and P(EDOT1-co-Pr1) with increasing sweep rates indicate a diffusion-controlled process [45]. Subsequently, the CV curves of the homopolymer and copolymer films obtained at a sweep rate of 200 mV s^−1^ were selected to find their corresponding redox peaks, and their associated anodic to cathodic peak-current ratios (I_pa_/I_pc_) were calculated; the results are shown in Table 2. The value of I_pa_/I_pc_ of the copolymer P(EDOT1-co-Pr2) was 1.10, which was closer to 1, and was in agreement with the observed reversibility of the redox peaks. The reversible redox behavior of the P(EDOT-co-Pr) copolymer indicates that it possesses excellent electrochemical properties.

The electrochemical responses at different sweep rates derived from the CV curves are displayed in Figure 5. The anodic and cathodic peak-current densities of the copolymer films gradually increased from 0.083 A/cm^2^ to 0.573 A/cm^2^ and from 0.060 A/cm^2^ to 0.520 A/cm^2^, respectively, when the sweep rate was increased from 25 mV s^−1^ to 300 mV s^−1^. This observation suggests that the copolymer film has a strong interfacial connection with the electrode and has good electrochemical activity [46]. Linear-fitting analysis of the peak-current density of the films revealed a linear correlation between the current density and the sweep rate, indicating that the film exhibits diffusion-controlled quasi-reversible behavior throughout the electrochemical process. The coefficient of determination (R^2^) for the linear fit of the anodic and cathodic peaks of the P(EDOT1-co-Pr1) copolymer films were 0.99414 and 0.99136, respectively, indicating excellent redox reversibility, adhesion, and electrochemical properties [47]. The corresponding R^2^ values for PEDOT and other copolymer films are also summarized in Table 2.

To study the diffusion characteristics of the PEDOT and P(EDOT-co-Pr) films during the electrochemical process, the ionic diffusion coefficient *D* of the films was calculated and analyzed. *D* directly quantifies the rates of ion injection and extraction within the electrochromic materials and is derived using Equation (1) [10].

(1) $$I_{p}=kn^{3/2}AD^{1/2}CV^{1/2}$$

where *I_p_* and *D* are the oxidation and reduction peak-current densities and ion diffusion coefficients in units of A/cm^2^ and cm^2^/s, respectively; *k* and *n* are the Randles–Sevcik constant and the number of exchanged electrons, where *n* is usually 1; and *A*, *C*, and *V* are the film area, the surface ion concentration, and the sweep rate in units of cm^2^, mol/cm^3^, and V/s, respectively. The surface ion concentration is converted from the electrolyte concentration. The cathodic and anodic polar diffusion coefficients (D_a_, D_c_) of the PEDOT and P(EDOT-co-Pr) films were calculated as shown in Table 2. The smaller the D_a_ value of the P(EDOT-co-Pr) film, the slower the charge injection rate of the copolymer films.

### 3.4. Spectroelectrochemical Characterization

**Figure 6:**
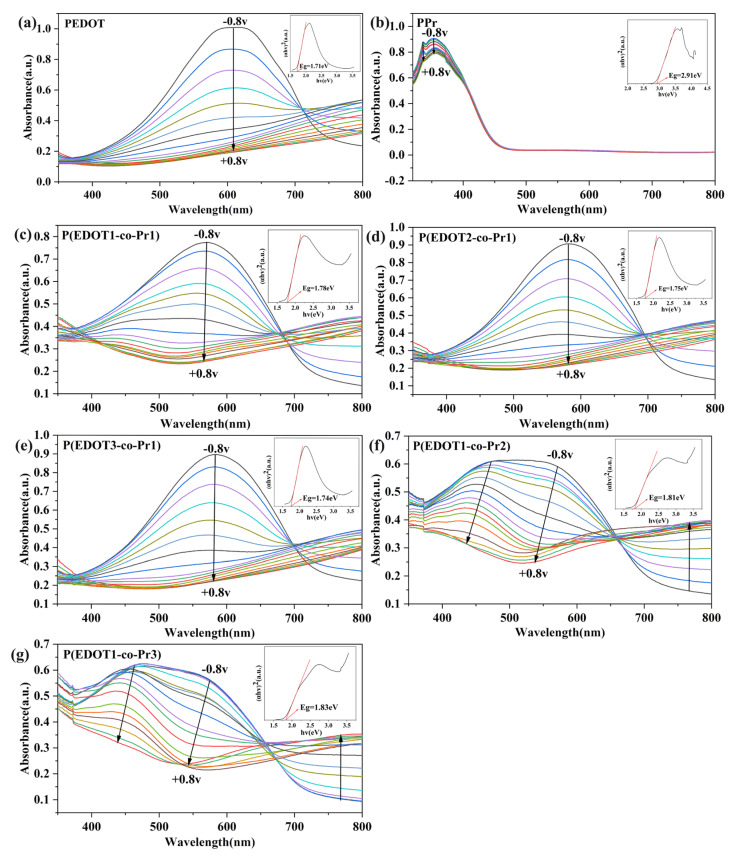
Spectroelectrochemistry of (**a**) PEDOT, (**b**) PPr, (**c**) P(EDOT1-co-Pr1), (**d**) P(EDOT2-co-Pr1), (**e**) P(EDOT3-co-Pr1), (**f**) P(EDOT1-co-Pr2), (**g**) P(EDOT1-co-Pr3)in LiClO_4_/PC (0.1 M) solution.

The spectroelectrochemistry of the homopolymer and copolymer films was examined by connecting an electrochemical workstation to a UV-Vis spectrophotometer. The UV-Vis spectra of the homopolymer and copolymer films in LiClO_4_/PC (0.1 M) solution at different potentials were examined in real time, and it was observed that the film spectra varied with the potential. As can be observed in Figure 6, the PPr film exhibits two distinct, intense absorption peaks centered at 331 and 354 nm, which is consistent with prior research findings [48]. The absorption spectral curves of the PPr film at different voltages do not show any significant change in the visible region, which indicates that it does not possess electrochromic properties. The P(EDOT1-co-Pr1) film shows a significant absorption peak at 564 nm, which is attributed to the π-π* electron jump in its reduced state [49]. It is noteworthy that the spectrum recorded across the iso-absorption point at 681 nm indicates that P(EDOT1-co-Pr1) is interconverting between its reduced and neutral states [25]. During the transition of the applied voltage from negative to positive, there is a substantial decrease in the peak intensity of the 564 nm absorption band, accompanied by the gradual emergence and intensification of a new absorption band centered around 781 nm. Polaron and bipolaron creation is thought to be the cause of this phenomenon [32]. The UV-Vis spectra of the PEDOT, P(EDOT2-co-Pr1), and P(EDOT3-co-Pr1) polymers show similar trends at different potentials.

The trend of the absorption peaks changes as the Pyrene in the copolymer films increases, and the P(EDOT1-co-Pr2) films have double absorption peaks at about 472 nm and 571 nm, which may be related to the vibrational coupling of the regionally symmetric polymer chains [50,51]. In addition, the π-π* transition absorption of P(EDOT1-co-Pr2) appears red-shifted during stepwise reduction, which may be related to the successive enhancement of the electronic conjugation by increasing the number of overlapping aromatic thiophene rings during the de-doping process [52]. During stepwise oxidation, although the π-π* electron-leaping band is weakening, a new transition occurs near 766 nm because of the formation of charge carrier bands. The copolymer P(EDOT1-co-Pr2) follows the same trend as P(EDOT1-co-Pr3). It is noteworthy that the copolymer P(EDOT-co-Pr) film shows a significant blue shift in the wavelength of the maximum absorption peak (λ_max_) in the reduced state when compared with the homopolymer PEDOT film. In addition, the UV-Vis spectra of the PEDOT, PPr, and P(EDOT-co-Pr) films show a significant shift in the bandwidth of the reduced state, which provides evidence of the successful synthesis of P(EDOT-co-Pr).

The optical bandgap (*E_g_*) is an important characteristic property of the homopolymer and copolymer films, which can be obtained by fitting the experimental absorption spectrum values with the Tauc [53] Formula (2).

(2) $${\left(\alpha hv\right)}^{n}=A\left(hv-E_{g}\right)$$

where *α* and *h* are the absorption coefficient and Planck’s constant, respectively; *ν* and *A* are the frequency and constant, respectively; and *n* is the Tauc coefficient (*n* = 2 for calculating the direct band gap).

As depicted in Figure 6, *E_g_* values were calculated by plotting (*αhν*)^2^ versus *hν* linearly for the films in the reduced state (−0.8 v), with *E_g_* determined by extrapolating the tangent line of the curve to the *x*-axis. The *E_g_* of 2.91 eV for the PPr film is in agreement with the results of a previous study [54]. It is noteworthy that the P(EDOT-co-Pr) copolymer films have a narrower range of optical bandgap (1.74–1.83 eV), which is explained by the presence of EDOT in the copolymer backbone, as this is able to mitigate steric hindrance and enhance molecular coplanarity, thus optimizing the structure and reducing the optical bandgap [55].

### 3.5. Electrochromic Properties

The electrochromic properties of the PEDOT, PPr, and P(EDOT-co-Pr) films were studied by analyzing the variations in their transmittance at specific wavelengths with time during rapid voltage switching of the homopolymer and copolymer films. In this research, the cycling stability, optical contrast (ΔT), coloration efficiency (CE), and switching time of the homopolymer and copolymer polymer films were determined and analyzed. The pertinent outcomes are succinctly presented in Table 3.

**Figure 7:**
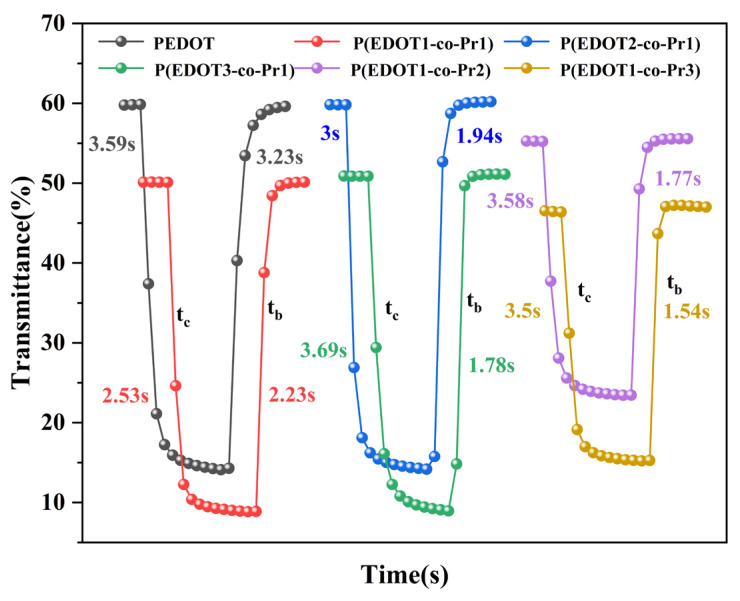
Switching response of the homopolymer and copolymers.

**Figure 8:**
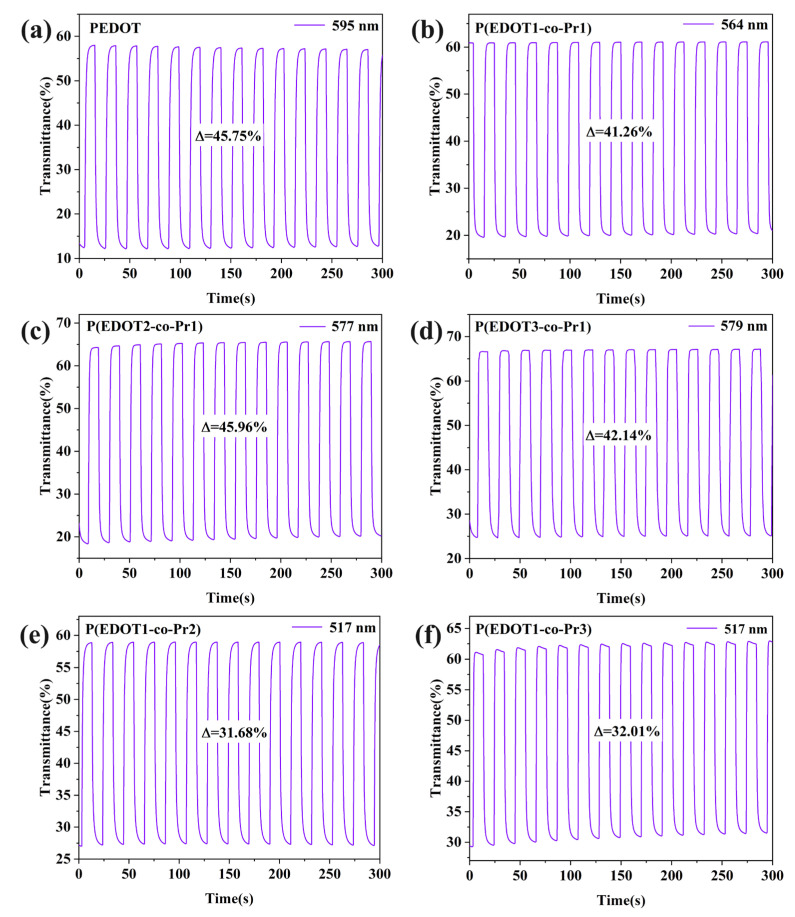
Optical contrast of (**a**) PEDOT, (**b**) P(EDOT1-co-Pr1), (**c**) P(EDOT2-co-Pr1), (**d**) P(EDOT3-co-Pr1), (**e**) P(EDOT1-co-Pr2), (**f**) P(EDOT1-co-Pr3).

Response time refers to the switching time between the coloration and bleaching states that is needed for a 95% change in the maximum optical contrast during the redox process [56]. The transmittance of the homopolymer and copolymer films was observed as a function of time by applying a potential of −0.6~+0.2 V with a switching time of 10 s in LiClO_4_/PC (0.1 M) solution at the corresponding λ_max_. Figure 7 and Figure 8 display the homopolymer and copolymer films’ response time and optical contrast curve plots of the redox cycling, respectively. The coloring time (t_c_) of the PEDOT film at 595 nm is 3.59 s, the bleaching time (t_b_) is 3.23 s, and the optical contrast is 45.75%. Meanwhile, the copolymer P(EDOT-co-Pr) films showed comparable or slightly lower optical contrast (31.68% to 45.96%). The bleaching process (1.54 s–2.23 s) was faster for copolymer P (EDOT-co-Pr) films compared with the PEDOT film.

**Figure 9:**
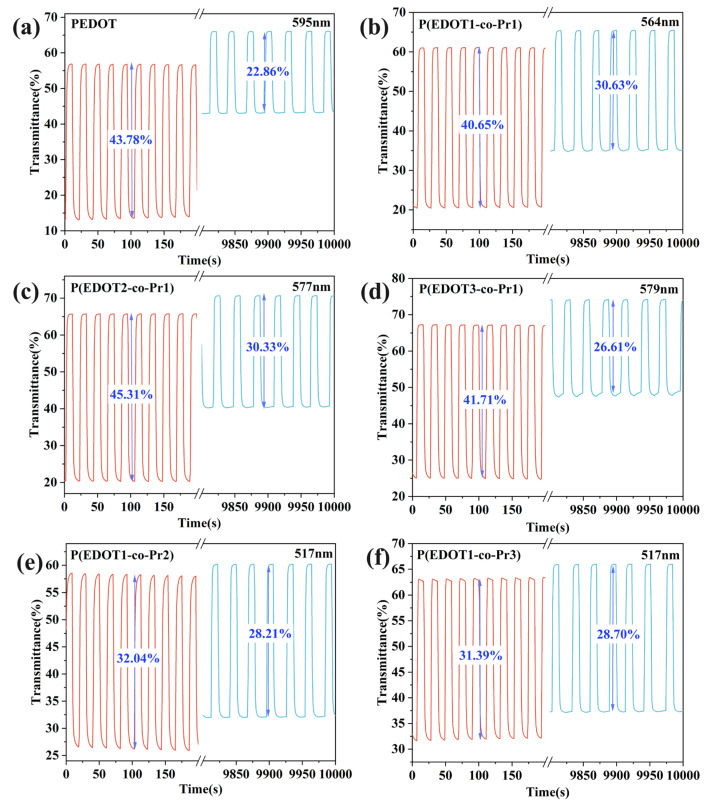
Cycling stability of (**a**) PEDOT, (**b**) P(EDOT1-co-Pr1), (**c**) P(EDOT2-co-Pr1), (**d**) P(EDOT3-co-Pr1), (**e**) P(EDOT1-co-Pr2), (**f**) P(EDOT1-co-Pr3).

Cyclic stability is an important performance index of electrochromic materials, which mainly reflects the maintenance of the discoloration performance of the material after many electrochromic cycles. The cycling stability of the electrochromic films was evaluated by connecting an electrochemical workstation with a UV spectrometer after several cycling tests. Figure 9a–f shows the cyclic stability of the polymer films measured by staying at switching voltages of −0.6 V and +0.2 V for 10 s. After cycling for 10,000 s, the homopolymer PEDOT maintained 52% of its initial optical contrast. The copolymer P(EDOT-co-Pr) retained a higher original optical contrast (64–91%). These data suggest that the P(EDOT-co-Pr) copolymer has superior cycling stability than the PEDOT homopolymer. This means that the P(EDOT-co-Pr) copolymer has a moderate and stable ion extraction rate during electrochromic processes.

One of the most critical measures of the effectiveness of electrochromic materials is their coloring efficiency (*CE*). The calculated *CE* provides a visual understanding of the extent and rate of change in the optical characteristics of the film under charge. The optical density difference (Δ*OD*) divided by the charge contained in the unit area (Δ*Q*) is the *CE* [47]. Typically, CE is derived utilizing Equation (3) as follows:

(3) $$CE=\Delta OD/\Delta Q=\log\left(T_{b}/T_{c}\right)$$

where Δ*OD* and Δ*Q* are the optical density change and charge per unit area of the electrochromic material during injection or withdrawal, respectively, and *T_b_* and *T_c_* are the transmittance during fading and coloring, respectively.

**Figure 10:**
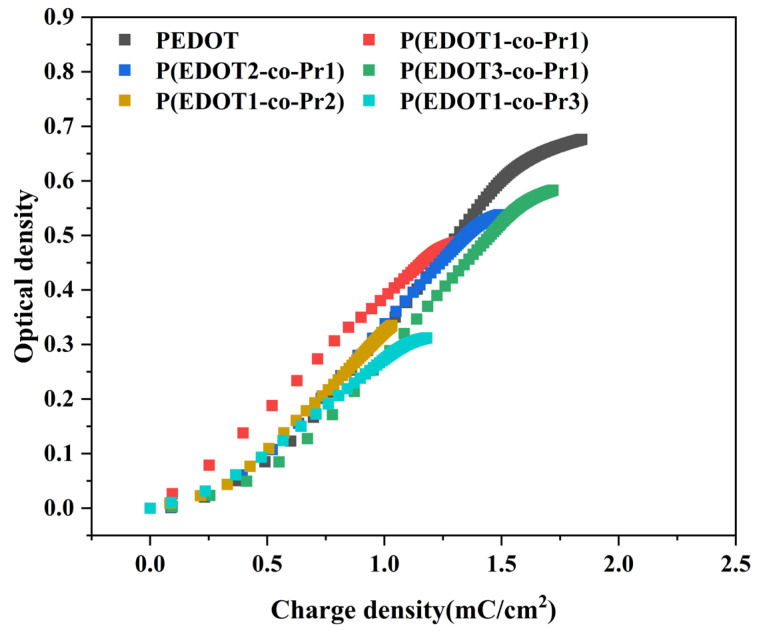
Coloring efficiency of the homopolymer and copolymers.

Electrochromic materials with a high *CE* typically show significant optical modulation when only a small amount of charge is injected (or withdrawn). The variation in Δ*OD* versus Δ*Q* for the copolymer P(EDOT-co-Pr) and the homopolymer PEDOT is shown in Figure 10. Based on the linear slope of the Δ*OD*/Δ*Q* curves, the PEDOT homopolymer has a coloring efficiency of 577 cm^2^ C^−1^. The coloring efficiency of the PEDOT homopolymer film is higher than that of the P(EDOT-co-Pr) copolymer films (350–507 cm^2^ C^−1^).

## 4. Conclusions

In this study, Pyrene was introduced into the EDOT chain and polymerized into copolymer P(EDOT-co-Pr) films by electrochemical polymerization, and the electrochemical, optical, and electrochromic properties of the films were investigated using CV, UV-Vis, and SEC. The copolymer P(EDOT-co-Pr) films with different monomer concentrations exhibit color shifts from pink-purple to dark green, light purple to light green, and blue-violet to blue-green, from light red to light brown to green, and from dark red to earthy yellow to green. The copolymer P (EDOT-co-Pr) has a low bandgap (1.74–1.83 eV), excellent optical contrast (31.68–45.96%), short bleaching time (1.54 s–2.23 s), and excellent cycling stability (64–91%). Among the copolymers, the P(EDOT1-co-Pr3) film exhibits an excellent bleaching process as well as outstanding cycling stability, maintaining 91% of the initial optical contrast after cycling for 10,000 s. It is thus a promising candidate copolymer for electrochromic devices.

## Figures and Tables

**Table 1:** The concentration and ratio of reagents.

Entry	Homopolymers/ Copolymers	Concentration of EDOT/Pyrene	Ratio of EDOT/Pyrene
(a)	PEDOT	10 mM	Neat EDOT
(b)	PPr	10 mM	Neat Pyrene
(c)	P(EDOT1-co-Pr1)	10 mM:10 mM	1:1
(d)	P(EDOT2-co-Pr1)	20 mM:10 mM	2:1
(e)	P(EDOT3-co-Pr1)	30 mM:10 mM	3:1
(f)	P(EDOT1-co-Pr2)	10 mM:20 mM	1:2
(g)	P(EDOT1-co-Pr3)	10 mM:30 mM	1:3

**Table 2:** Anodic to cathodic peak-current ratio, linear-fitting coefficients, and ion diffusion coefficients of the homopolymer and copolymers films.

Homopolymer/ Copolymers	I_pa_/I_pc_	R^2^ (cathode)	R^2^ (anode)	D_c_ (cathode)	D_a_ (anode)
PEDOT	1.3	0.99069	0.99914	3.29 × 10^−11^	9.46 × 10^−11^
P(EDOT1-co-Pr1)	1.13	0.99136	0.99414	4.08 × 10^−11^	6.14 × 10^−11^
P(EDOT2-co-Pr1)	1.14	0.98954	0.99266	4.76 × 10^−11^	8.01 × 10^−11^
P(EDOT3-co-Pr1)	1.14	0.99594	0.99598	4.87 × 10^−11^	8.15 × 10^−11^
P(EDOT1-co-Pr2)	1.10	0.99407	0.99317	3.78 × 10^−11^	5.31 × 10^−11^
P(EDOT1-co-Pr3)	1.11	0.98681	0.98515	3.36 × 10^−11^	4.83 × 10^−11^

**Table 3:** Electrochromic properties of the homopolymer and copolymer films.

Homopolymers/ Copolymers	λ_max_ (nm)	E_g_ (eV)	T% (%)	Response Time		CE (cm^2^·C^−1^)	Optical Stability (%)
				t_c_ (s)	t_b_ (s)		
PEDOT	595	1.71	45.75	3.59	3.23	577	52
PPr	331/354	2.91	/	/	/	/	/
P(EDOT1-co-Pr1)	564	1.78	41.26	2.53	2.23	456	75
P(EDOT2-co-Pr1)	577	1.75	45.96	3	1.94	507	67
P(EDOT3-co-Pr1)	579	1.74	42.14	3.69	1.78	506	64
P(EDOT1-co-Pr2)	472/571	1.81	31.68	3.58	1.77	461	88
P(EDOT1-co-Pr3)	462/574	1.83	32.01	3.5	1.54	350	91

## Data Availability

The original contributions presented in this study are included in the article/Supplementary Materials. Further inquiries can be directed to the corresponding author.

## Supplementary Materials

The following supporting information can be downloaded at: https://www.mdpi.com/article/10.3390/polym17010069/s1, Figure S1: CV curves of (a) P(EDOT2-co-Pr1), (c) P(EDOT3-co-Pr1), (e) P(EDOT1-co-Pr2) and (g) P(EDOT1-co-Pr3) films in monomer-free LiClO_4_/PC (0.1 M) solution at a 25 to 300 mV s^−1^ sweep rate; Electrochemical response of (b) P(EDOT2-co-Pr1), (d) P(EDOT3-co-Pr1), (f) P(EDOT1-co-Pr2) and (h) P(EDOT1-co-Pr3) films; Figure S2: Photos of PEDOT, PPr, P(EDOT1-co-Pr1), P(EDOT2-co-Pr1), P(EDOT3-co-Pr1), P(EDOT1-co-Pr2), and P(EDOT1-co-Pr3) in the reduced and oxidized states (−0.8 v and +0.8 v, respectively); Video S1: PEDOT color change in the range of −0.8 to +0.8; Video S2: P(EDOT1-co-Pr1) color change in the range of −0.8 to +0.8; Video S3: P(EDOT2-co-Pr1) color change in the range of −0.8 to +0.8; Video S4: P(EDOT3-co-Pr1) color change in the range of −0.8 to +0.8; Video S5: P(EDOT1-co-Pr2) color change in the range of −0.8 to +0.8; Video S6: P(EDOT1-co-Pr3) color change in the range of −0.8 to +0.8.
