# Supplementary figures and images for: Improving the Electrochemical and Electrochromic Properties of Copolymerized 3,4-Ethylenedioxythiophene with Pyrene
Source: Polymers (Basel). 2024 Dec 30;17(1):69. doi: 10.3390/polym17010069 (PMC11722773; doi:10.3390/polym17010069)

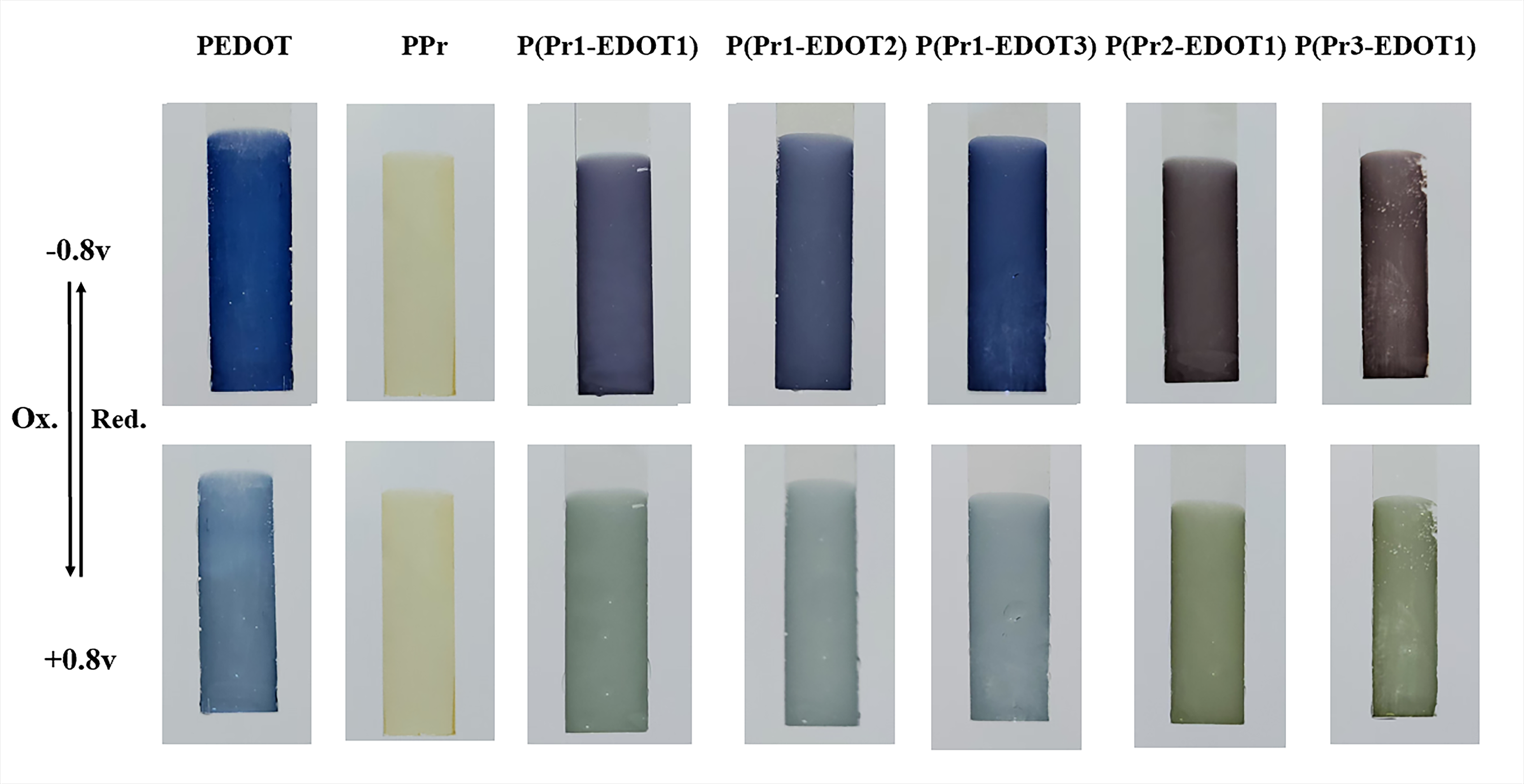

Supplement: Supplementary file 1 [file polymers-17-00069-s001.zip › Figure S2.tif]
